# Case Report: Focal xanthogranulomatous pyelonephritis in children: diagnostic pitfalls and the role of conservative management

**DOI:** 10.3389/fneph.2025.1709724

**Published:** 2025-11-18

**Authors:** Bochra Aziza, Nada Sghairoun, Nader Bennour Ghaddab, Yasmine Houas, Said Jlidi

**Affiliations:** Department of Pediatric Surgery, Children’s Hospital of Tunis, Tunis, Tunisia

**Keywords:** pyelonephritis, xanthogranulomatous, pediatric, surgery, conservative treatment

## Abstract

**Background:**

Focal xanthogranulomatous pyelonephritis (XGP) is a rare chronic renal inflammatory disorder in children that often mimics renal neoplasms, complicating diagnosis and management.

**Methods:**

We describe two pediatric cases of focal XGP managed at our institution and provide a descriptive review of the literature (1975–2024), analyzing clinical presentation, imaging features, management strategies, and outcomes of this disease.

**Results:**

Case 1: A 2-year-old boy presented with a febrile right flank mass and systemic inflammation. CT Scan revealed an 80 mm multilocular renal mass. Surgical drainage and biopsy confirmed focal XGP, and targeted antibiotics led to complete resolution with preserved renal function at two-year follow-up. Case 2: A 10-year-old girl presented with a 40 mm left renal mass and systemic inflammatory signs. CT-guided aspiration and histopathology confirmed focal XGP. She was managed conservatively with intravenous and oral antibiotics, achieving complete resolution and normal renal function at seven-year follow-up. Literature review of 34 pediatric XGP cases (median age 11.1 years) showed that 53% were focal lesions. Conservative management with antibiotics, with or without drainage, succeeded in 64% of cases, and overall outcomes were favorable, with stable renal function and no reported mortality.

**Conclusion:**

This combined case series and descriptive literature review highlights that conservative, kidney-sparing management is a feasible and effective approach in selected pediatric focal XGP cases. Multicenter collaborations are needed to define standardized diagnostic and therapeutic protocols.

## Introduction

Xanthogranulomatous pyelonephritis (XGP) is a rare chronic renal inflammatory disorder marked by parenchymal destruction and replacement with lipid-laden, macrophage-rich, granulomatous tissue (1). Despite characteristic histopathology, focal XGP often presents as a renal mass, creating diagnostic uncertainty between inflammatory and neoplastic lesions in children. Few pediatric cases have been reported, and optimal management remains undefined.

This study aims to describe two pediatric cases of focal XGP and review the literature to assess diagnostic challenges and the feasibility of conservative, kidney-sparing management.

## Materials and methods

We retrospectively analyzed two pediatric cases of focal xanthogranulomatous pyelonephritis (XGP) managed at Bechir Hamza Children’s Hospital, Tunis. Data reviewed included demographics, clinical presentation, laboratory and imaging findings, intraoperative details, and histopathology.

A systematic descriptive literature search was performed to identify pediatric cases of xanthogranulomatous pyelonephritis (XGP) published between 1975 and 2024 in PubMed, Embase, and Google Scholar using terms related to “xanthogranulomatous pyelonephritis,” “children,” “pediatric,” and “renal infection.” Studies were included if they reported histopathologically confirmed focal or diffuse XGP in patients <18 years, with details on clinical presentation, imaging, management, and outcomes. Exclusion criteria were adult-only studies, reviews without case data, and non-English/French publications without extractable information. Titles and abstracts were screened independently by two reviewers, with full texts assessed for eligibility and discrepancies resolved by consensus. Data were extracted on patient demographics, lesion type, diagnostics, management, outcomes, and follow-up, and summarized in **Table 1**; study selection is illustrated in a PRISMA flow diagram (Figure X).

**Table 1:** Summary of published pediatric XGP cases (1975-2024).

Author (Year)	n	Age (years)	Sex/side	Urine culture	Imaging findings (CT/US)	Preoperative diagnosis	Conservative treatment (antibiotics ± drainage, duration)	Surgical treatment	Histopathology (ANA-PATH)	Type
Deng QF et al., 2022 (15)	1	7	R	*S. aureus*	Heterogeneous cystic lesion	Renal abscess	Antibiotics × 15 days	Partial nephrectomy	Foamy macrophages, granulomatous inflammation	Focal
Marteinsson VT et al., 1995 (16)	1	6	L	Candida albicans	Avascular mid-portion mass	Renal abscess	Antibiotics × 3 months	Partial nephrectomy	XGP with fungal colonies	Focal
Nam JK et al., 2011 (10)	14	9 (mean)	R=10, L = 3, B = 1	Proteus mirabilis (4), E. coli (1)	Pyelonephrosis, renal enlargement, calculi	Renal cell carcinoma	Not specified	Total nephrectomy (13), Partial (1)	Granulomatous inflammation, lipid-laden macrophages	Focal (9), Diffuse (5)
López-Medina A et al., 1994 (8)	1	9	R	No growth	Upper pole mass with perirenal extension	Renal tumor	No	Radical nephrectomy	Chronic granulomatous inflammation	Focal
Shah K et al., 2012 (4)	1	0.5	L	E. coli	Diffuse cystic mass	Renal cyst	No	Radical nephrectomy	Granulomatous inflammation with giant cells	Diffuse
Gupta S et al., 2009 (17)	1	17	L	Mixed flora	Poorly enhancing mass with calculi	Pyonephrosis	IV antibiotics × 4 days + nephrostomy drainage	Radical nephrectomy	Diffuse granulomatous inflammation	Diffuse
Shanser JD et al., 1975 (18)	1	4	L	No growth	Renal mass with perinephric thickening	Renal tumor/abscess	No	Local excision + drainage	Xanthogranulomatous inflammation	Focal
Shah K et al., 2011 (19)	1	12	R	No growth	Bilateral mid-pole lesions with septations	Renal tumor	Antibiotics × 3 months	None	Chronic granulomatous inflammation	Focal
Yazaki T et al., 1982 (20)	1	3	R	Klebsiella pneumoniae	Enlarged kidney with pyonephrosis	Pyonephrosis	Antibiotics + nephrostomy	Radical nephrectomy	Lipid-laden macrophages, necrosis	Focal
Cakmakci H et al., 2002 (13)	1	14	R	E. coli	Homogeneous hypoechoic mass, bilateral hydroureteronephrosis	Renal tumor	No	Radical nephrectomy	Diffuse granulomatous process	Diffuse
Hoeffel JC et al., 1998 (21)	1	6	R	No growth	Enlarged, heterogeneous renal mass	Renal tumor	Chemotherapy + antibiotics	Radical nephrectomy	Diffuse granulomatous inflammation	Diffuse
Morais CG et al., 2022 (7)	1	0.1	L	No growth	Progressive nodular lesion (mid-third)	XGP	Antibiotics × 7 weeks	None	Focal granulomatous inflammation	Focal
Nadeem M et al., 2013 (22)	1	14	L	Proteus mirabilis, E. coli	Hypodense renal mass	Renal tumor	No	Radical nephrectomy	Xanthogranulomatous inflammation	Focal
Nandedkar SS et al., 2014 (6)	1	8	L	No growth	Ill-defined mass with perirenal extension	Wilms tumor	No	Radical nephrectomy	Foamy macrophages and necrosis	Focal
Ding X et al., 2020 (23)	1	4	R	No growth	Infiltrative renal mass	RCC mimic	No	Partial nephrectomy	Granulomatous inflammation	Focal
Iumanne S et al., 2016 (9)	1	3	L	No growth	Multicystic lesion with renal pelvis destruction	Renal abscess/tumor	No	Radical nephrectomy	Foamy macrophages	Focal
Julson JR et al., 2022 (24)	1	14	R	No growth	Renal enlargement	Renal tumor	No	Radical nephrectomy	Xanthogranulomatous inflammation	Focal

Nb*: number of patients, R*: right, L*: left.

## Results

### Case presentation

#### Case 1

A 2-year-old boy presented with a 4-day history of fever (38.8°C), right flank pain, and a palpable tender mass. On examination, blood pressure was 95/60 mmHg (50th percentile for age), heart rate 110 bpm, and temperature 38.8 °C. Laboratory investigations showed leukocytosis (18,200/mm³; ref. 4,000–10,000), anemia (Hb 10.5 g/dL), and elevated inflammatory markers (CRP 65 mg/L; ref. < 5 mg/L; ESR 52 mm/h). Urinalysis demonstrated pyuria (15–20 WBC/HPF) and trace proteinuria; urine culture grew *Escherichia coli* sensitive to third-generation cephalosporins. Blood culture was sterile. Serum creatinine was 0.36 mg/dL (ref. 0.2–0.5 mg/dL), and estimated glomerular filtration rate (eGFR) calculated by the Schwartz formula was 110 mL/min/1.73 m².

**Figure 1:**
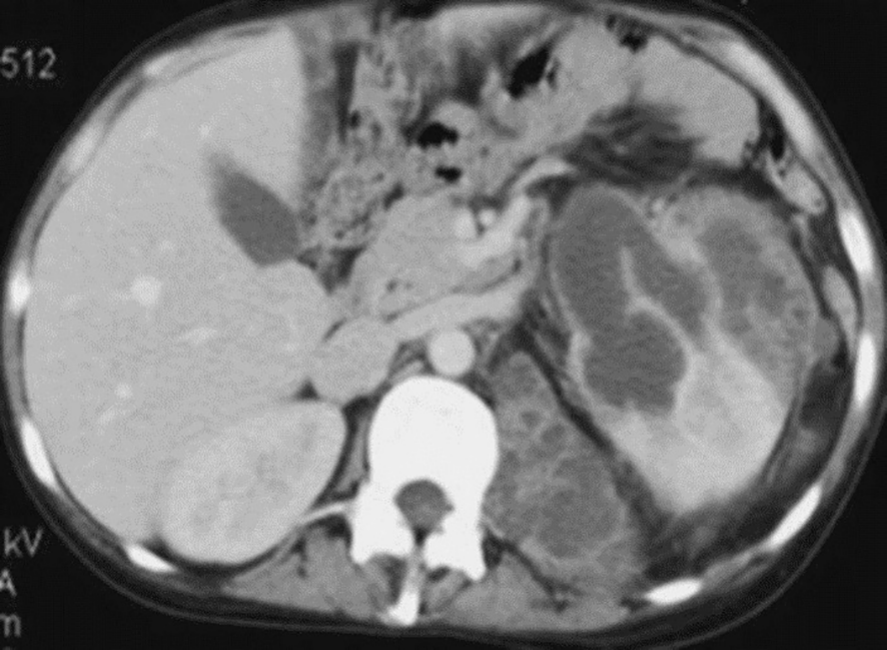
Contrast-enhanced abdominal CT scan showing a multilocular right lower-pole renal mass (white arrow). Scale bar = 1 cm.

Ultrasonography revealed a multilocular hypoechoic mass in the lower pole of the right kidney. Contrast-enhanced CT (venous phase) showed an 80 mm multiloculated, peripherally enhancing lesion with perirenal fat stranding (**Figure 1**), suggesting either an abscess or neoplasm. Due to diagnostic uncertainty, surgical exploration via a para-rectal approach was performed, revealing a large abscess adherent to adjacent structures. Drainage and biopsy were done. Histopathology confirmed focal xanthogranulomatous pyelonephritis, with sheets of foamy macrophages and granulomatous inflammation. Targeted intravenous antibiotics (cefotaxime + amikacin + metronidazole) were administered for 10 days, followed by oral therapy for three weeks. IV therapy was maintained until the patient was afebrile for 48 hours and CRP decreased by >50%. Follow-up ultrasonography at 6 weeks and 2 years confirmed complete resolution with preserved renal parenchyma and stable renal function (eGFR 110 mL/min/1.73 m²).

#### Case 2

**Figure 2:**
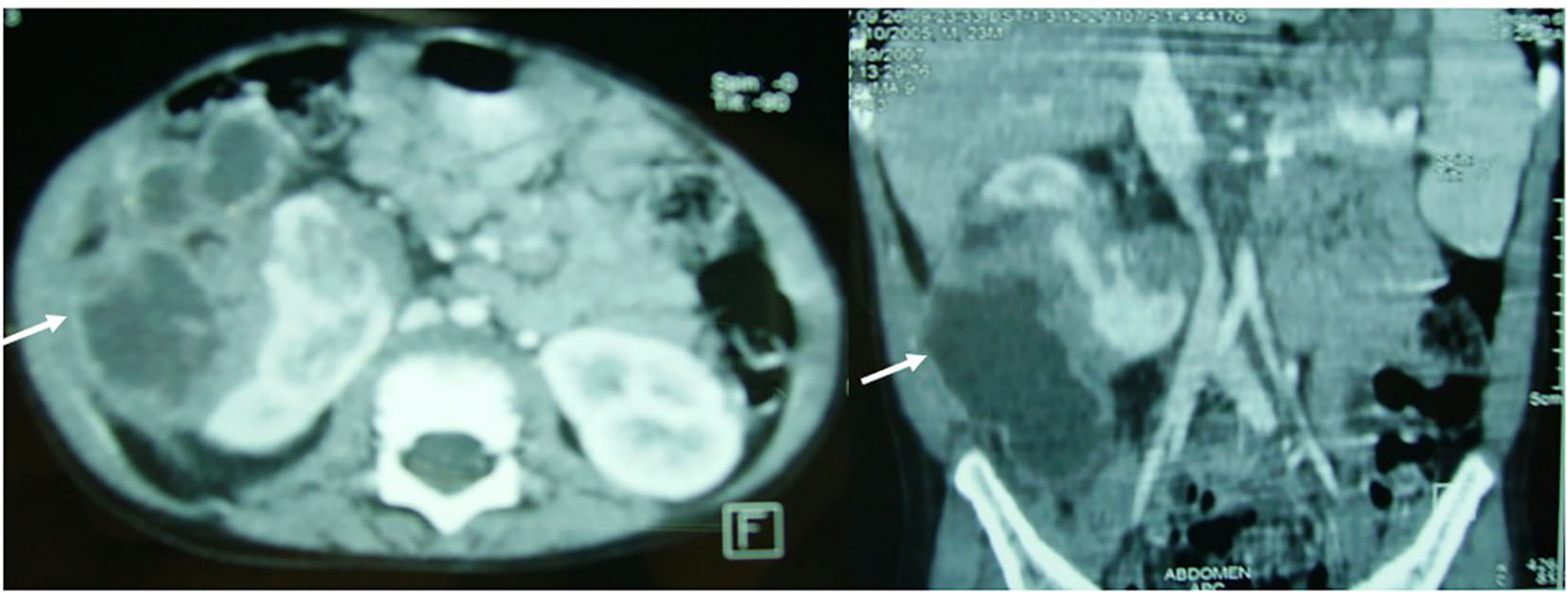
Abdominal CT scan showing a well-demarcated, multiloculated left mid-renal mass with peripheral rim enhancement (white arrow). *Scale bar = 1 cm.

A 10-year-old girl presented with intermittent fever (38.5 °C), malaise, left flank pain, and a 2 kg weight loss over one month. Physical examination revealed a tender left flank mass; blood pressure was 100/65 mmHg (60th percentile). Laboratory results showed leukocytosis (16,200/mm³), anemia (Hb 9.8 g/dL), and elevated CRP (110 mg/L). Urinalysis demonstrated pyuria (10–15 WBC/HPF), but urine and blood cultures were sterile, likely due to prior antibiotic use. Serum creatinine was 0.52 mg/dL (ref. 0.3–0.6 mg/dL), eGFR 98 mL/min/1.73 m². Ultrasonography revealed a 40 mm heterogeneous mass in the mid-zone of the left kidney. CT (venous phase) showed a well-defined multiloculated lesion with peripheral rim enhancement (**Figure 2**), consistent with an early suppurative process. CT-guided aspiration yielded purulent material; cultures were sterile, but histopathology confirmed focal XGP. The patient received a 14-day course of intravenous cefotaxime, amikacin, and metronidazole, followed by 4 weeks of oral cephalosporin. Criteria for switching to oral therapy were clinical improvement, defervescence, and >50% CRP reduction. Follow-up imaging at 3 months showed complete regression. At 7-year follow-up, renal function and growth parameters remain normal (eGFR 98 mL/min/1.73 m²) (**Table 2**).

**Table 2:** Clinical timeline of the two patients.

Case	Age	Event	Diagnostic/therapeutic intervention	Outcome
1	2 years	Fever + right flank mass	Labs: WBC: 18,212/mm³ (NR 4,500–13,500), CRP: 65 mg/L (NR <5)	Clinical suspicion
	2 years	Imaging	US + CT: 80 mm multilocular renal mass	Indeterminate (inflammatory vs tumor)
	2 years	Surgery	Para-rectal exploration, abscess drainage, biopsy	Histopathology focal XGP, *E. coli* culture
	2 years	Antibiotics	third-generation cephalosporin, aminoglycoside (3 days), and metronidazole IV 10 days → Oral 3 weeks	Resolution, preserved kidney
	4 years	Follow-up	Imaging + labs	Normal renal function (eGFR 110 mL/min/1.73 m²)
2	10 years	Fever + left flank mass	Labs: WBC: 16,200/mm³ (NR 4,500–13,500), CRP: 110 mg/L (NR <5), Hb: 9.8 g/dL	Clinical suspicion
	10 years	Imaging	US + CT: 40 mm mid-renal mass	Indeterminate
	10 years	CT-guided aspiration	Biopsy + culture (sterile)	Histopathology focal XGP
	10 years	Antibiotics	third-generation cephalosporin, aminoglycoside (3 days), and metronidazole IV 14 days → Oral 4 weeks	Resolution, preserved kidney
	17 years	Follow-up	Imaging + labs	Normal renal function (eGFR 98 mL/min/1.73 m²), no recurrence

## Discussion

Xanthogranulomatous pyelonephritis (XGP) is a rare, destructive, and chronic renal inflammatory disease (2). The initial description of the pathologic features of XGP was published in 1916 by Schlagenhaufer (3). Histologically, it is characterized by replacement of the renal parenchyma with granulomatous tissue containing lipid-laden macrophages, which impart the typical yellow coloration. The condition is usually associated with persistent urinary tract infections and chronic obstruction, most often due to nephrolithiasis (4). Two morphological forms are recognized: diffuse (92%) and focal (8%), the latter frequently mimicking renal carcinoma (3). Although most commonly reported in middle-aged women, pediatric cases are rare but documented. In children, XGP typically occurs before the age of 8, with a male predominance, and often presents as a focal, localized, and acalculous form (5). Clinical manifestations are usually nonspecific, including fever, flank pain, and urinary tract infection (4). Laboratory findings often reveal leukocytosis, anemia, and elevated Erythrocyte Sedimentation Rate (1). Urine cultures may identify a range of organisms, with Escherichia coli and Proteus mirabilis being the most common pathogens (6, 7). In Case 2, urine culture was sterile, likely due to empirical antibiotics initiated before referral. Sterile cultures are not uncommon in XGP and may obscure pathogen identification. Absence of bacterial growth should prompt broad initial antimicrobial coverage and careful clinical monitoring, as treatment response, not culture results, guides therapeutic duration.

Because of its variable clinical and radiological features, the preoperative diagnosis of XGP is difficult and often inaccurate. While ultrasonography and other imaging modalities may suggest XGP, CT and MRI remain the most reliable investigations. In focal cases, CT typically reveals a well-defined intrarenal mass with fluid-like attenuation and rim enhancement, often mimicking a renal tumor (8). This makes preoperative diagnosis particularly challenging in children, and many cases are confirmed only after surgical exploration (9). Management depends on disease extent. Diffuse involvement generally necessitates nephrectomy, as the kidney is usually nonfunctional due to extensive parenchymal destruction (10–12). By contrast, focal XGP in children has increasingly been managed conservatively. Several reports describe successful outcomes with prolonged antibiotic therapy, sometimes combined with percutaneous drainage, thereby preserving renal tissue (13). Antibiotic regimens are typically culture-directed, with broad-spectrum agents initiated empirically and treatment often extended for weeks to months, guided by clinical improvement, inflammatory markers such as ESR or CRP, and serial imaging. When surgery is required, nephron-sparing approaches are preferred over nephrectomy to optimize renal preservation (14). Our two pediatric cases further support conservative therapy as a valid alternative in selected patients, particularly when disease is localized, renal function is preserved, obstruction is absent or relieved, and the child remains clinically stable. Renal function was monitored by serum creatinine and estimated glomerular filtration rate (eGFR) calculated using the Schwartz formula. Both patients maintained normal renal function at last follow-up (final eGFR 110 mL/min/1.73 m² and 98 mL/min/1.73 m², respectively), providing objective evidence supporting kidney-sparing management. Review of published pediatric series indicates that successful conservative outcomes are associated with localized disease, preserved renal architecture, and the absence of obstructive uropathy, whereas diffuse XGP generally necessitates nephrectomy due to non-functioning parenchyma. Several case series and reviews have also reported encouraging results with antibiotics alone, segmental resection, or local debridement, further supporting the role of kidney-sparing strategies in focal XGP. These observations emphasize the importance of early radiologic recognition and careful case selection for conservative management. However, significant challenges remain: preoperative differentiation from renal malignancy is often difficult, standardized antibiotic protocols and treatment durations are not well established, and data on long-term outcomes, including recurrence, hypertension, and renal impairment, remain limited.

**Table 1** summarizes the clinical features, the imaging modalities, the number of cases confirmed preoperatively and the surgical management report across the included studies.

## Conclusion

Focal XGP in children poses significant diagnostic challenges due to its similarity to renal tumors. Our experience and literature review demonstrate that conservative, kidney-sparing management can be effective in carefully selected cases, provided close clinical and imaging follow-up is ensured. Future multicenter collaborations are needed to define standardized diagnostic and therapeutic protocols for pediatric focal XGP.

## Data Availability

The original contributions presented in the study are included in the article/supplementary material. Further inquiries can be directed to the corresponding author.
